# Noise-resilient exceptional point sensing with immunity to undesired perturbations

**DOI:** 10.1126/sciadv.aeb7018

**Published:** 2026-02-27

**Authors:** Serena Landers, William Tuxbury, Ilya Vitebskiy, Tsampikos Kottos

**Affiliations:** ^1^ Wave Transport in Complex Systems Lab, Department of Physics, Wesleyan University, Middletown, CT 06459, USA.; ^2^ Sensors Directorate, Air Force Research Laboratory, Wright Patterson AFB, Dayton, OH 45433, USA.

## Abstract

Exceptional point degeneracies (EPDs) are non-Hermitian singularities where *K* eigenvalues and their corresponding eigenvectors coalesce. When a small perturbation is induced, the eigenvalue detuning from an EPD follows a *K*th-root sublinear expansion, which provides a means of enhancing the sensitivity (frequency shift) of resonant-based sensors. On the downside, resonant-based sensors are susceptible to cavity imperfections, local mechanical disturbances (temperature variations, vibrations), and other experimental uncertainties. Here, we overcome this problem by experimentally implementing passive periodic microwave metamaterials with nonresonant EPDs (NR-EPDs) occurring in their Bloch spectrum. We demonstrate a sublinear variation of the reflectance near NR-EPDs to a specific class of (global) perturbations and propose its usage for ultrasensitive sensing that is immune to undesired (local) perturbations. The sensitivity is shielded from technical or fundamental noise that typically degrades the signal-to-noise performance of resonant EPDs.

## Teaser

Nonresonant exceptional points enable ultrasensitive sensing that is robust to noise and immune to undesired local imperfections.

## INTRODUCTION

High-sensitivity sensors are critical for a variety of military and civilian applications ranging from dead reckoning in GPS-denied or GPS-inadequate situations and noise-prone mechanical gyroscopes in smartphones, to structural integrity monitoring, wearable health trackers, and internet of things (IoT) sensors for the detection of environmental changes (*1*–*9*). Typical sensors detect small perturbations by measuring their effects on a physical observable with a linear response (LR) to the perturbation. Abandoning the LR approach opens up qualitatively different opportunities in high precision sensing. A prominent example is resonant systems operating near a *K*th-order exceptional point degeneracy (EPD), where a small perturbation ε ≪ 1 activates a sublinear response in the resonant splitting $\Delta f\propto \sqrt[{K}]{\varepsilon }\gg \varepsilon$, due to the fractional Puiseux expansion of the perturbed resonances around the EPD (*10*) (see, also (*7*–*9*, *11*–*14*) and references therein). The implementation of the resonant-based EPD sensing led to the development of a class of EPD-based sensors such as biosensors (*15*, *16*), gyroscopes (*17*–*19*), accelerometers (*20*, *21*), implant wireless respiration (*22*) and intracranial pressure (*23*) sensors, surface-acoustic-wave gas sensors (*24*), and more. At the same time, the hypersensitive nature of resonant EPD sensors is also the Achilles heel of these devices: They are very sensitive to local mechanical disturbances (e.g., vibrations) and cavity imperfections originating from fabrication errors. Therefore, it will be extremely useful for practical sensing applications to advance different design concepts that decouple the effects of fabrication errors and experimental uncertainties from perturbations caused by measurements.

The efficiency of EPD-based sensors has been further scrutinized on the basis of their resonant resolvability and their signal-to-noise ratio (SNR) figure of merit (*18*, *25*–*32*). In the case of purely lossy EPD-based sensors, the former is limited by the broadening of the resonance linewidths. This deficiency can be rectified by introducing gain; however, this generates (technical) noise that degrades the SNR performance of the sensor. Furthermore, the eigenbasis collapse at the EPD introduces an additional source of fundamental noise, which contributes to linewidth broadening. Studies have argued that this noise can counteract the sensitivity enhancement near EPDs, thus resulting in an SNR that is not exceptional but rather conventional (*18*, *26*–*28*).

**Fig. 1.**
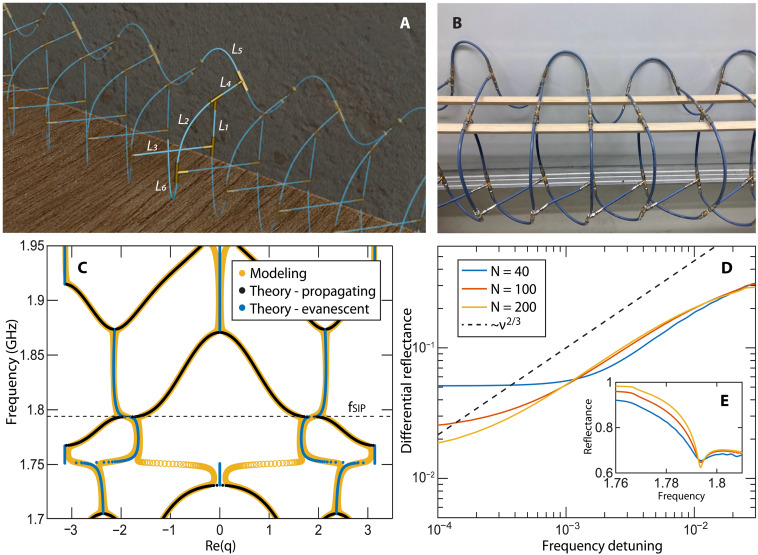
Experimental platform of the nonresonant, SIP-based sensor, its Bloch dispersion relation, and scaling analysis of the differential reflectance. (**A**) Experimental SIP platform with the defining coaxial cable lengths $l_{1},\ldots ,l_{6}$ of one unit cell indicated. (**B**) Photograph of the network array, showing only a portion of the unit cells. The coaxial cables that make the SIP periodic meta-structure are supported by two (wooden rods). (**C**) Dispersion relation of the periodic network of (A) and (B) in the absence of any ohmic losses. The black (blue) filled circles indicate the propagating (evanescent) band (gap). The open yellow circles indicate the Bloch modes in case of absorption. The SIP frequency at $f_{\text{SIP}}\approx 1.79$ GHz is indicated with a dashed horizontal line. (**D**) The scaling of the differential reflectance $\Delta R\left(f\right)\equiv |R\left(f\right)-R_{0}|$ versus the frequency detuning $\nu \equiv |f-f_{\text{SIP}}|$ from the SIP frequency for three different network sizes corresponding to *N* = 40 (blue line), 100 (red line), (yellow line) unit cells. Network losses are considered. (**E**) The reflectance spectrum R(f) of the network of (A) and (B) in proximity of the SIP frequency for the three different network sizes [the same color coding as in (D)] and in the presence of ohmic losses. The position of $f_{\text{SIP}}$ remains unaffected by the size of the network and by the presence of losses.

Here, we experimentally demonstrate an EPD-based sensing protocol that alleviates the susceptibility to mechanical disturbances and unintentional fabrication errors unrelated to the measured physical observable. The protocol maintains a desirable sublinear response, which ensures high responsivity to a specific family of global, system perturbations while simultaneously mitigating issues associated with resolvability and noise enhancement. As opposed to resonant-based schemes, our design involves passive periodic metamaterials that feature EPDs in the Bloch spectrum of transfer matrices (*33*–*37*). The transfer matrix is an inherently nonnormal operator and, therefore, can naturally exhibit EPDs in its spectrum without the necessity to implement gain or loss elements in the meta-structure. We focus our attention on the engineering of a third-order EPD (EPD-3), which results in the formation of a stationary inflection point (SIP) in the passband of the Bloch dispersion relation, where the group velocity of propagating modes vanishes. Artifacts from the SIP are reflected in the transport observables of finite scattering samples, which can serve as sensing measurands—distinct from the resonant splitting Δ*f* that is typically used in resonant EPD protocols. Specifically, we define the differential reflectance as $\Delta R\equiv |R\left(f\right)-R_{0}|$, the absolute deviation of the measured reflectance R(f) from a reference value $R_{0}$ extracted from the background spectrum (see below). This quantity exhibits a characteristic sublinear dependence on a global detuning parameter ε from the SIP conditions, i.e., $\Delta R∼\varepsilon^{2/3}$. We experimentally demonstrate this behavior for a periodic network of microwave coaxial cables (see Fig. 1, A and B) that support an SIP in its Bloch dispersion relation (Fig. 1C). We show that for $\varepsilon =\nu =f-f_{\text{SIP}}$, where $f_{\text{SIP}}$ is the SIP frequency, the differential reflectance scales as $\Delta R∼\nu^{2/3}$; see Fig. 1D. We experimentally demonstrate that this sublinear response scaling is robust to structural imperfections, such as fabrication errors, owing to the nonresonant nature of this kind of EPD. Furthermore, the sensitivity χ=∂ΔR/∂ε is not plagued by fundamental noise due to resonant mode collapse, nor does it suffer from technical noise due to the presence of active elements (the structure is completely passive). Instead, we find that the proposed sensor has two-order of magnitude–enhanced SNR in the proximity of the EPD-3.

The proposed sensing scheme is integrated circuit (IC) compatible, compact, requires low-power budget, and has minimal maintenance costs. In addition to avionic sensing (accelerometers, inclinometers, gyroscopes, etc.), it can be used in a variety of sensor applications, such as noninvasive corrosion and crack detection, nondestructive evaluation of structural integrity, platform stabilization for space applications, and intruder detection.

## RESULTS

### Two important SIP-related properties

There are two attributes associated with SIPs that are attractive for realizing ultrasensitive sensors with imperfection tolerant and noise-resilient characteristics (*33*). These properties are platform independent, relying on generic properties of SIPs, and manifest in the transport spectra of finite samples. As we will show below, they are rooted in the cubic scaling relation between frequency $\nu \equiv f-f_{\text{SIP}}$ and wave number $\delta k\equiv k-k_{\text{SIP}}$ detuning in proximity to an SIP

$$\nu ∼\delta k^{3}$$ (1)

where the cubic exponent signifies the formation of an EPD-3 in the Bloch spectrum of the transfer matrices.

#### Sublinear response

When incident monochromatic waves of fixed flux $S_{I}$ are injected into an extended semi-infinite, lossless structure supporting an SIP (with a frequency of the incident signal being in the proximity of $f_{\text{SIP}}$), the scaling of transmitted amplitude $a_{T}$ of the slow propagating wave depends on the number of evanescent modes $n_{e}$ that are excited by the impinging wavefront (*33*–*36*)

$$a_{T}∼{\delta k}^{-n_{e}}$$ (2)

Using Eq. 1, we can show that its flux $S_{T}$ transmitted inside the sample—which is proportional to the product of the group velocity $v_{g}=\partial \omega /\partial k∼\nu^{2/3}$ (ω=2πf is the angular frequency) and the intensity of the transmitted wave $I∼{|a_{T}|}^{2}$—has the possibility to exhibit sublinear scaling if $n_{e}=0$ evanescent modes are excited because of the specific shape of the incident wavefront (see the Supplementary Materials). Under these ideal scattering conditions, i.e., when a specific wavefront that does not engage evanescent modes is injected into a lossless, semi-infinite SIP structure, the transmitted flux at frequencies that are detuned from the SIP frequency by $\nu =|f-f_{\text{SIP}}|$ scales as (*33*–*36*)

$$S_{T}∼\nu^{2/3}$$ (3)

indicating that for ν→0, we have $S_{T}=0$ and therefore the reflectance and transmittance at $f=f_{\text{SIP}}$ are $R\left(f_{\text{SIP}}\right)=1\;\text{and}\;T\left(f_{\text{SIP}}\right)=0$, respectively. At the same time, we have that R(f)+T(f)=1, which results in the following expression for the differential reflectance: $\Delta R\left(\nu \right)\equiv |R\left(f_{\text{SIP}}+\nu \right)-R\left(f_{\text{SIP}}\right)|=S_{T}/S_{I}∼S_{T}$. In other words, under the above ideal conditions, the differential reflectance develops a cusp at $f_{\text{SIP}}$ which is characterized by the sublinear scaling indicated by Eq. 3, thus allowing us to use this measurand for sublinear response sensing protocols (*33*). The sublinear response simultaneously provides enhanced sensitivity for small variations, while also enhancing the dynamic range of the sensor due to the reduced sensitivity away from the SIP point. While the above argumentation assumed that ν is the frequency detuning from the SIP frequency, the same sublinear scaling applies for any detuning ε of a global system parameter, e.g., a global variation of the magnetic field penetrating the periodic structure (*33*).

**Fig. 2.**
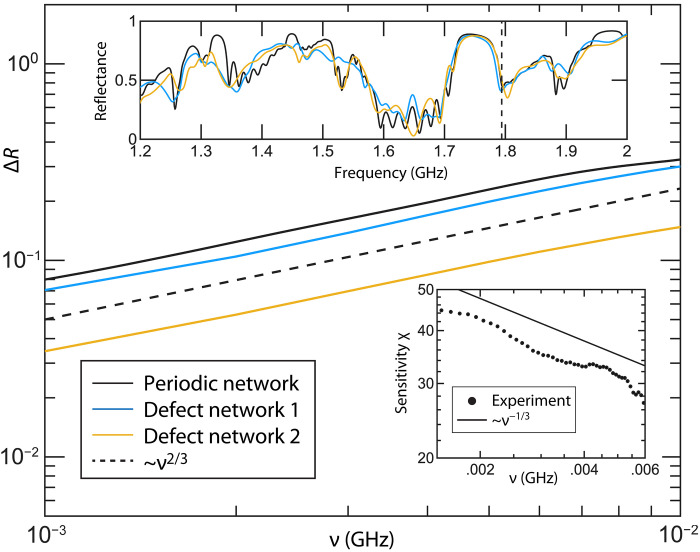
Sensitivity of the nonresonant, SIP-based sensor. Measured differential reflectance Δ*R* with respect to frequency detuning ν from the SIP frequency. Solid black line indicates the experimental results measured from a periodic network. Solid blue and yellow lines indicate the experimental results for two different configurations of “defective” networks where three randomly chosen unit cells have been distorted by removing a randomly chosen coaxial cable from each (see details in Materials and Methods). The dashed black line indicates the theoretically predicted power-law scaling $\Delta R\propto \nu^{2/3}$. Top, inset: Measured reflectance spectrum *R*(*f*) versus frequency *f* for the periodic (black line) and the two defective network samples (light blue and yellow lines). The SIP frequency is marked with a dashed line. Bottom, inset: Sensitivity χ versus frequency detuning ν extracted from the differential reflectance measurements of the periodic network.

**Fig. 3.**
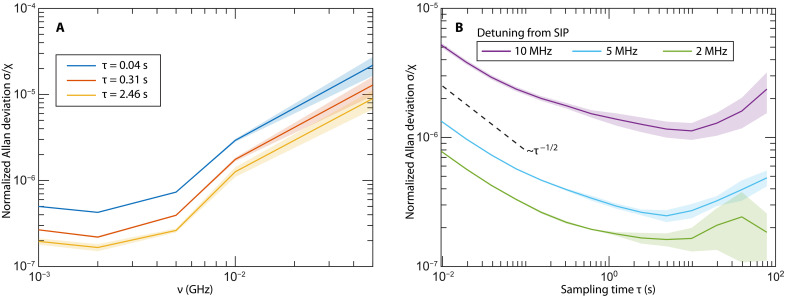
Noise analysis of the nonresonant, SIP-based sensors. (**A**) Normalized Allan deviation for the perfect network at several sampling times τ(s) versus the frequency detuning from the SIP. (**B**) Normalized Allan deviation for the periodic network as a function of sampling time for three different detunings being in the proximity (ν=2 and ν=5 MHz) and away (ν=10 MHz) from the SIP frequency. The dashed black line indicates an inverse power law $1/\sqrt{\tau }$ associated with random walk noise due to additive noise.

Any realistic SIP structure has finite size and hence gives rise to Fabry-Perot resonances observable in the reflection spectra. These oscillations can pollute the scaling indicated by Eq. 3. Fortunately, this predicament can be mitigated by the presence of losses in the structure, which broadens the resonance line widths so that the oscillations in the reflection are diminished. While the presence of losses disrupts the ideal SIP conditions, the sublinear response is still preserved in proximity to the EPD-3. However, extremely close to the EPD-3 losses wash out the reflectance cusp while they also reduce its value from unity. Therefore, $R\left(f_{\text{SIP}}\right)$ of the ideal extended structure cannot be measured directly. In addition, imperfections in the shape of the injected wavefront (e.g., due to the finite size of the sample) can partially excite evanescent modes, thus further distorting the reflectance extremely close to $f_{\text{SIP}}$ (see the Supplementary Materials). In our sensing protocol, we consider such effects originating from the nonideal scattering conditions and extract the reference reflectance $R_{0}$ by extrapolating the background reflectance spectrum R(f) toward $f_{\text{SIP}}$. The best fit power law that has been used for the background reflectance in both simulations (Fig. 1D) and measurements (Figs. 2 and 3) is $R=R_{0}+\alpha {\left(f-f_{\text{SIP}}\right)}^{2/3}$, and was implemented over the range of the sensor to extract the reference reflectance $R_{0}$, which defines the measurand, $\Delta R\left(\nu \right)\equiv |R_{0}-R\left(\nu \right)|$ that has been used in practice (for a detailed discussion see the Supplementary Materials and fig. S1). It is important to point out that the extracted $R_{0}$ from the measured data (or simulations) using the above scheme characterizes uniquely the (imperfect) scattering process associated with the specific sensing platform near the SIP. Consequently, the sensing measurand ΔR(ν) that is used to evaluate the size of the perturbations is also defined uniquely for the specific structure. The robustness of the differential reflectance sublinear response to nonideal scattering conditions (*33*, *38*–*40*) is demonstrated in Fig. 1D where we have simulated the differential reflectance ΔR versus the frequency detuning from the SIP frequency ν of the finite periodic network of Fig. 1 (A and B) in the presence of ohmic losses. The latter are modeled via an imaginary part of the wave number (see below and Materials and Methods for further details). Notice that the dynamical range of the sensor, the domain where the sublinear response is maintained, stretches closer to the SIP frequency as the loss is progressively reduced for larger system sizes *N*.

#### Robustness

The second attribute of an SIP-based sensor is its robustness to local imperfections, which is justified by the following argument. Wave quantization results from the particular boundary conditions of a problem and is encoded by the wave number *k*. Therefore, local variations occurring in a periodic sample (e.g., length of the sample or the presence of defects) can be interpreted as small perturbations to the effective wave number. Then, Eq. 1 indicates that the effect of local perturbations are cubically suppressed in the vicinity of the SIP frequency. Therefore, aberrations to the ideal structure will not strongly distort scaling properties associated with the SIP, for example, Eq. 3.

### The microwave network platform and its theoretical modeling

A key design requirement for a periodic structure that supports an SIP in its Bloch dispersion is that the unit-cell transfer matrix must have dimensions higher than three. This condition enables the emergence of a threefold degeneracy among the Bloch eigenmodes of the transfer matrix. Equally important for the structure is to maintain as few symmetries as possible (apart, of course, from translational symmetry). Another important element is the presence of multiple pathways connecting adjacent unit cells, which generate strong interference between counterpropagating waves and thereby facilitate the formation of slow propagating waves. Similar design principles have been used theoretically in earlier studies using network models and discrete coupled-mode theory models that demonstrate SIPs (*36*, *39*–*42*). Our experimental SIP platform, designed to demonstrate the efficiency of the proposed sensing scheme, has been inspired by these considerations. It consists of a periodic network of coaxial cables, where each unit cell comprises six varying lengths $\left(l_{\alpha };\alpha =1,\ldots ,6\right)$ connected by tee junctions (valency 3) at vertices of the cell. Each unit cell incorporates four vertices arranged to provide intercell and intracell vertex connectivity that ensures multiple propagating pathways. This architecture resulted in a unit-cell transfer matrix of dimension 6 × 6, sufficient to support an SIP degeneracy.

Specifically, the network is formed from 15 repeated unit cells, where each unit cell consists of four tee junctions (μ=1,…,4). There are three interior connections within each unit cell: Cable of length $l_{1}$ connects vertex μ=1 with vertex μ=2, cable of length $l_{2}$ connects vertex μ=2 with vertex μ=3, and cable of length $l_{4}$ connects vertex i=2 with vertex μ=4. There are also three connections between adjacent unit cells: Cable of length $l_{3}$ connects vertex μ=3 of unit cell *n* with vertex μ=1 of unit cell n+1, cable of length $l_{6}$ connects vertex μ=1 of unit cell *n* with vertex μ=3 of unit cell n+1, and cable of length $l_{5}$ connects vertex μ=4 of unit cell n with vertex μ=4 of unit cell n+1 (see Fig. 1A). To avoid any symmetries (apart from the translational symmetry due to periodicity), care was taken that these lengths are not rationally related.

A theoretical modeling of the periodic network of Fig. 1 (A and B) is formulated by assuming single mode operation in each cable, subject to the Helmholtz equation (see details in Materials and Methods). By imposing appropriate boundary conditions at each vertex, i.e., the solution must satisfy wave continuity (voltage) and flux conservation (current) at each vertex, it is possible to establish a matrix formulation of the problem

M(ω=2πf)∣ϕ⟩=0 (4)

where $|\phi \rangle ={\left[\ldots ,|\phi_{n-1}\rangle ,|\phi_{n}\rangle ,|\phi_{n+1}\rangle ,\ldots \right]}^{T}$ is an infinite dimensional vector encoding the wave amplitude at each of the vertices and $|\phi_{n}\rangle$ is a 4 × 1 vector encoding the wave amplitude at each of the vertices in the *n*^*th*^ unit cell. The dispersion relation associated with the periodic structure can be computed by considering the four equations of Eq. 4 associated with the *n*^*th*^ unit cell. Since there are only nearest neighbor interaction between unit cells, **M** has a block tridiagonal form so the equations of the *n*^*th*^ unit cell are of the form

$$M_{n,n-1}|\phi_{n-1}\rangle +M_{n,n}|\phi_{n}\rangle +M_{n,n+1}|\phi_{n+1}\rangle =0$$ (5)

where $M_{n,m}$ is the $\left(n^{\mathrm{th}},m^{\mathrm{th}}\right)$ 4 × 4 sub-block of **M**. Equation 5 can be used to express the transfer matrix of the unit cell (see Materials and Methods for details). The latter takes the form

$$T_{n}\dot{=}\left(\begin{array}{cc} -M_{n,n+1}^{-1}M_{n,n} & -M_{n,n+1}^{-1}M_{n,n-1}\\ I_{4} & 0 \end{array}\right)$$ (6)

where $I_{4}$ is the 4 × 4 identity matrix. The eigenvalues $\lambda_{\beta }\;\left(\beta =1,\ldots ,8\right)$ of the transfer matrix are related to the Bloch wave numbers $q_{\beta }a=-i\;\text{ln}\lambda_{\beta }$, providing the Bloch dispersion relation by plotting $\left[f,\text{Re}\left(q_{\beta }a\right)\right]$. Bloch modes corresponding to real $q_{\beta }$ are identified as propagating modes while complex $q_{\beta }$ indicate evanescent modes. The lengths of the cables, $l_{\alpha }$, are then tuned to support an SIP at the desired frequency *f* ≈ 1.79 GHz. An evaluation of the Bloch dispersion relation for the periodic network of Fig. 1 (A and B) is shown in Fig. 1C. The black (blue) dots indicate the propagating (evanescent) branches in case of a lossless network. In the same figure, we also report with yellow dots the dispersion relation for a lossy network where the ohmic losses of the coaxial cables have been evaluated from the experiment and modeled by an imaginary part in the index of refraction (see Material and Methods).

The sample is turned to a scattering setup by truncating the matrix **M** to *N* unit cells, which we denote **M**_*N*_, and coupling three transmission lines (TLs) at the front end of the structure. The wave in the TLs also satisfies continuity at the vertices where it is coupled

$$|s^{-}\rangle =-|s^{+}\rangle +W^{T}|\phi \rangle$$ (7)

where $|s^{\pm }\rangle$ are 3 × 1 vectors containing the incoming/outgoing wave amplitudes from the TLs, **W** is a 4*N* × 3 binary matrix describing the connection between the TLs and the respective vertices, and ∣ϕ⟩ is a 4*N* × 1 vector containing the wave amplitudes at the vertices inside the array. The steady-state equations of the open system can be written

$$\left(M_{N}+iW^{T}W\right)|\phi \rangle =2iW^{T}|\phi \rangle$$ (8)

Together, Eqs. 7 and 8 can be used to derive the scattering matrix of the sample

$$S=-{𝕀}_{3}+2i\text{WG}W^{T}$$ (9)

where the resolvent is $G={\left(M_{N}+iW^{T}W\right)}^{-1}$. From the scattering matrix we can extract the total reflectance $R=\sum_{m=1}^{3}{|S_{m3}|}^{2}$ versus the incident frequency and evaluate the scaling of the differential reflectance Δ*R* with respect to its SIP value. In Fig. 1D, we show these results for three different system sizes consisting of *N* = 40, 100, 200 unit cells. We have chosen an incident wavefront that is orthogonal to the eigenstate associated with the minimal eigenvalue of the reflectance matrix $S^{†}\left(f_{\text{SIP}}\right)S\left(f_{\text{SIP}}\right)$ of the finite structure (which efficiently couples to the slow mode; see also Materials and Methods). Notice that **S** is not unitary because of ohmic losses in the coaxial cables (see Materials and Methods). The observed saturation of the differential reflectance Δ*R* for extremely small detuning values ν is associated with the presence of these losses and the finite size of the structure. Nevertheless, as *N* is increased while decreasing loss such that the total absorption remains approximately constant, the sublinear response domain where $\Delta R∼\nu^{2/3}$ stretches further to smaller detuning values. The position of the SIP frequency remains unaffected by the increased size of the scattering sample (see Fig. 1E), indicating that the SIP is not a resonant phenomenon (*33*–*35*, *38*).

### Sensitivity measurements and noise analysis

Scattering experiments were conducted on the coaxial network consisting of *N* = 15 unit cells. The scattering parameters were collected by directly connecting 3 channels (*m* = 1, 2, 3) of a VNA source to vertices μ = 1, μ = 4, and μ = 3 of the first unit cell, respectively. Corresponding vertices on the opposite end of the structure were terminated using 50 Ω impedance loads. Multiple sets of measurements were performed on variants of the array with impurities intentionally built into the structure to demonstrate the extreme robustness inherent to SIP phenomena. Last, the noise resilience, attributed to the non-resonant nature of the NR-EPD sensor, was verified via an analysis of Allan deviation which was evaluated for various frequency detunings away from the SIP.

#### Sensitivity analysis of an SIP sensor and robustness to local imperfections

The “ideal” experimental array is constructed without intentional defects from *N* = 15 identical unit cells as described in the previous section with a precision of approximately 0.1 mm in the physical length of each cable. The total reflectance $R=\sum_{m=1}^{3}{|S_{m3}|}^{2}$ due to an input signal to channel *m* = 3, was collected in the 1- to 2-GHz range, with a clear signature of the SIP occurring at the frequency *f* ≈ 1.79 GHz predicted by numerical evaluation of the dispersion relation (see Materials and Methods).

The choice of the incident wavefront was selected on the criteria that the reflectance exhibits a sublinear scaling with respect to detuning from the SIP frequency, indicated by Eq. 3. The sublinear response of the measured differential reflectance Δ*R* is reported in Fig. 2 as a black solid line. The theoretical prediction $\Delta R\propto \nu^{2/3}$, is also shown at the same figure with a black dashed line. Implementation of a finite difference method to the experimental data allows us to evaluate the sensitivity $\chi \equiv \frac{\partial \Delta R}{\partial \nu }$ (see black-dotted line in the lower inset of Fig. 2), which in the proximity of the SIP scales with the detuning ν as $\chi ∼\nu^{-1/3}$ (see solid black line in the lower inset of Fig. 2). The sensitivity increase saturates as we move toward smaller detunings ν. This saturation in the sensitivity is associated with the small number of unit cells that we have used in our experiment and can be further mitigated by increasing *N* (see Fig. 1D). For larger values of the detuning the power law scaling of the sensitivity with the detuning becomes more abrupt because of the influence of the nearby band-edge and the extreme high-Q resonances that populate this domain (*34*, *35*).

To demonstrate the robustness of SIP-based sensors to fabrication errors, the structure was intentionally altered by removing three cables from the structure entirely, with 50-Ω loads terminating the ends of the tee junctions from which they were removed. The three defect unit cells from which a cable was removed have been randomly chosen, and we have confirmed that the results remain qualitatively the same for other choices of unit cells. In the upper inset of Fig. 2, we report the measured reflectance spectrum of two defect structures (light blue and yellow lines) together with the spectrum measured from the periodic (perfect) network (solid black line). The defect configuration 1 (light blue line) represents a typical scenario. while the defect configuration 2 (yellow line) describes the most extreme perturbation of our network where the removed cable was at the closest distance from the injected boundary. It is apparent that the reflectance from the deformed networks closely matches the results of the perfect structure in the proximity to the SIP frequency, while the behavior of the reflectance at other frequency ranges is strongly affected by the alteration of the cavity.

Measurement of the differential reflectance Δ*R* versus the frequency detuning ν was collected for the defect systems (light blue and yellow solid lines), and the results are reported in the main panel of Fig. 2 together with the measurements from the perfect network (black solid line). From our data, it is clear that even with the defect unit cells present, the sublinear response scaling properties of the differential reflectance are preserved in proximity to the SIP frequency. The small variation of the reflectance spectrum near the SIP frequency occurring in the second defect configuration (yellow line) does not affect at all the scaling of the differential reflectance.

The robustness of the EPD-3 to local imperfections distinguishes our proposal from all existing EPD-sensing schemes. As we discussed above, the physical origin of this robustness is traced back to the nonresonant nature of SIPs.

#### Noise analysis

Next, we turn to the noise analysis of the signal for the proposed sensor. To this end, we use the Allan deviation that describes the stability of the sensor as a function of the sampling time τ (*43*–*45*). The Allan deviation σ(τ) of the differential reflectance Δ*R* is defined as $\sigma \left(\tau \right)=\sqrt{\frac{1}{2\left(L-1\right)}\sum_{n=1}^{L-1}{\left[\langle \Delta R^{\left(n+1\right)}\rangle -\langle \Delta R^{\left(n\right)}\rangle \right]}^{2}}$, where τ is the sampling time, *L* is the total number of reflectance measurements, and $\langle \Delta R^{\left(n\right)}\rangle$ indicates the average differential reflectance during the sampling time interval [nτ,(n+1)τ]. Repeated measurements were performed for 20,000 iterations at fixed frequencies away from the SIP frequency over the course of 192.01 s to characterize the Allan deviation associated with the noise present in the sensor. The Allan deviation is normalized by the sensitivity χ and is reported in Fig. 3A with respect to detuning from the SIP frequency for three sampling times. The SD of the measurements is highlighted by the shaded outline of the curves and becomes negligible close to the SIP frequency. From the figure, it can be seen that the noise, relative to the sensitivity, is dramatically reduced (by almost two orders) in the proximity of the SIP frequency.

In Fig. 3B, we report the rescaled Allan deviation versus the sampling time τ for three representative values of the detuning. The smallest two detuning values are well within the sublinear response domain associated with the SIP. In particular, the short-time behavior of the Allan deviation (random walk noise due to displacement read-out noise, or additive noise from the source and electronics used in the measurements) is the most relevant for sensing purposes and behaves like $\sigma /\chi ∼1/\sqrt{\tau }$. We have experimentally demonstrated that the proximity to the NR-EPD3 does not affect the value of σ indicating that the noise in the measured signal Δ*R* is not enhanced and therefore, the SNR σ/χ is dominated completely by the sensitivity enhancement in the proximity of the SIP.

## DISCUSSION

We have experimentally demonstrated an enhanced sensing protocol that exploits nonresonant EPDs occurring in the spectra of transfer matrices of passive periodic meta-structures. As opposed to resonant EPD sensing schemes that exploit the sublinear resonant detuning of resonant frequencies as the sensing measurand, our protocol uses the sublinear response of the differential reflectance when a specific class of perturbing agents interact with the system. Because reflectance is a directly measurable observable, it is less susceptible to the uncertainties associated with any resonance-shift sensing schemes (including both standard and resonant EPD sensing), which require inferring the resonant position from experimentally acquired observables e.g., reflected/transmitted intensities or amplitudes. Such processes are often challenging because of a linewidth broadening of the resonances and the error-prone task of fitting spectral line shapes. Introduction of gain elements might enforce a narrowing of the linewidth, but it also results to the generation of additional noise that deteriorates the SNR. In addition, resonant shift sensing involves broadband frequency sweeps that can be time-consuming and impose an upper bound on the dynamical range.

Using a microwave metamaterial consisting of a periodic network of coaxial cables, we show that our NR-EPD sensor mitigates the problems associated with noise enhancement (due to gain or eigenmode collapse) that plague the resonant EPD sensors. It demonstrates robustness to a large class of undesired perturbations associated with mechanical disturbances or structural imperfections that are unrelated to the measured physical observable, thus, decoupling the effects of fabrication errors and other experimental uncertainties from perturbations caused by measurements and establishing the proposed NR-EPD scheme for practical sensing applications.

Qualitatively similar approaches to achieving Bloch dispersion relations supporting an SIP can and have been realized at optical frequencies; see for example (*46*) and references therein. The approach in (*46*) and other similar studies is also based on periodic arrays of coupled optical waveguides. The problem, though, is that at optical frequencies, such an approach has severe fundamental limitations related to strong radiative losses. The way to suppress radiative losses is to make a unit cell of the periodic array much larger compared to the optical wavelength, implying that the operational frequency falls into a high-order photonic band. The latter results in extreme proximity of SIP to other spectral features, such as the photonic band edges. As a consequence, even a tiny perturbation, such as structural imperfections, frequency variations, or nonlinearity, would result in dramatic changes in light propagation and scattering, which puts severe limitations on this approach at optical wavelengths. By contrast, at microwave frequencies, the problem of radiative losses is virtually nonexistent. This allows us to work in a low-order photonic band, where the separation of the frozen mode frequency from the nearest photonic band edges is substantial, and we can clearly separate the effects related to the frozen mode regime from those related to other spectral singularities.

That said, at optical wavelengths, there is an alternative approach based on a periodic chain of defects in a two-dimensional (2D) photonic structure [see, for example, (*47*)]. The operational frequency of such a linear chain of defects falls into a photonic bandgap of the 2D periodic array, which suppresses the radiative losses and makes it possible to work within a low-order photonic band of the periodic chain of defects. In principle, using this approach, the results of our microwave study could be replicated at optical wavelengths.

The proposed microwave or optical SIP-based sensing platform could consist of a compact local resonant probe coupled to a remote SIP-enabled transduction stage. The probe can be any high-Q optical or microwave resonator whose eigenfrequency responds to a physical perturbation; representative implementations include optomechanical Fabry-Perot cavities or microwave LC resonators, where an applied acceleration displaces a test mass and alters the cavity length or capacitor-plate spacing, respectively, as well as whispering gallery–mode structures whose resonance shifts under rotation via the Sagnac effect [see for example Fig. 1 in (*33*)]. The perturbation-induced resonance shift transforms a broadband incident waveform into a narrowband signal centered at a detuned frequency. This signal is subsequently directed to a periodic metamaterial array engineered to support an SIP degeneracy, where the reflected field exhibits the characteristic cusp-like response associated with an SIP. Tracking the resulting differential reflectance variations ΔR provides a robust, sublinear, defect-immune, and noise-resilient readout of the imposed perturbation and constitutes the primary measurand of the sensing protocol. The SIP protocol can be used in a variety of applications ranging from avionics and temperature variation sensing to bio- and chemical sensing.

## MATERIALS AND METHODS

### Experimental details

The microwave network was constructed according to the connectivity described in the “microwave network platform and its theoretical modeling” section, where each bond is made from coaxial cables (model S_04272_B), cut to the specified lengths within 0.1-mm accuracy, and attached at SMA Tee adapters (FFF) via SMA coaxial cable plug adapters. All open junctions and unterminated cable ends were capped with 50-Ω SMA termination loads. The physical cable lengths, accounting for the lengths of the plugs are, $l_{1}=126.3$ mm, $l_{2}=211.6$ mm, $l_{3}=114.4$ mm, $l_{4}=147.3$ mm, $l_{5}=180.3$ mm, and $l_{6}=181.0$ mm. The system was probed using Keysight Streamline Vector Network Analyzer model P5023B. The real part of the refractive index was obtained by measuring the transmission phase shift Δϕ of individual cables using $n^{\prime }=\frac{c\;\Delta \phi }{2\pi \mathrm{fl}}$, where *f* is the incident frequency, *c* the speed of light, and *l* the physical cable length.

To demonstrate robustness of the platform, two “defective” configurations were tested with three cables removed entirely. The removed cables in the first configuration were $l_{5}$ between unit cells 5 and 6, $l_{2}$ in unit cell 10, and $l_{1}$ in unit cell 3 (light blue line in Fig. 2). In the second configuration, the removed cables were $l_{1}$ in unit cell 12, $l_{3}$ between unit cells 6 and 7, and $l_{6}$ between unit cells 2 and 3 (yellow line in Fig. 2).

### Graph modeling

Microwave graph networks consist of coaxial cables (bonds) connected at junctions (vertices), where the wave is subject to continuity and current conservation boundary conditions. In this section, we derive the generic scattering formulation used for modeling the slow wave chain. The formulation provides an analytical description of the system by expressing its equations of motion in a compact matrix form, using the field amplitudes on the vertices as a basis.

The wave amplitude along each bond ψ_μβ_ (connecting vertices μ and β and of length *l*_μβ_) is assumed to satisfy the 1D wave equation

$$\frac{d^{2}}{dx^{2}}\psi_{\mu \beta }\left(x\right)+\varepsilon_{r}{\left(\frac{\omega }{c}\right)}^{2}\psi_{\mu \beta }\left(x\right)=0;x\equiv x_{\mu \beta }\in \left[0,l_{\mu \beta }\right]$$ (10)

where the relative permittivity along each bond ε_*r*_ is assumed to be constant. The variable *x*_μβ_ indicates the position along the bond from vertex μ to vertex β; however, to avoid redundant notation, the subscript will often be suppressed when it can be implied by another context, e.g., ψ_μβ_.

The system can be turned to a scattering set-up by connecting interrogating leads to vertices of the graph. The wave amplitude in the lead connected to vertex μ (hereon simply referred to as lead μ) due to an incoming wave amplitude *I*_α_ in lead α (indicated by the superscript) is

$$\psi_{\mu }^{\left(\alpha \right)}=I_{\alpha }\delta_{\mu \alpha }e^{-\mathrm{ikx}}+\Gamma_{\mu \alpha }e^{\mathrm{ikx}};x\in \left(-\infty ,0\right]$$ (11)

where Γ_μα_ is the reflected/transmitted wave amplitude in lead μ due to an input wave from lead α. Denoting the wave amplitude at a vertex μ as $\phi_{\mu }^{\left(\alpha \right)}$, the continuity boundary condition at vertex μ can be formulated in the bonds and leads respectively as

$$\phi_{\mu }^{\left(\alpha \right)}=\psi_{\mu \beta }^{\left(\alpha \right)}\left(x=0\right);\phi_{\mu }^{\left(\alpha \right)}=\psi_{\mu }^{\left(\alpha \right)}\left(x=0\right)$$ (12)

Similarly, the current conservation boundary conditions at vertex μ can be formulated as

$$\sum_{\beta }{\left[A\right]}_{\mu \beta }\frac{d\psi_{\mu \beta }^{\left(\alpha \right)}}{\mathrm{dx}}{|}_{x=0}=\left\{ \begin{array}{c} -\frac{d\psi_{\mu }^{\left(\alpha \right)}}{\mathrm{dx}}{|}_{x=0},\;\text{if vertex}\;\mu \;\text{has}\;a\;\text{lead connection, }\\ 0,\;\mathrm{else} \end{array}\right.$$ (13)

where **A** is the adjacency matrix, which encodes the graph connectivity

$${\left[A\right]}_{\mu \beta }=\left\{ \begin{array}{c} 1,\;\text{if vertices}\;\mu \;\text{and}\;\beta \;\text{share}\;a\;\text{bond}\\ 0,\;\text{else}\;\left(\text{including}\;\mu =\beta \right) \end{array}\right.$$ (14)

Taking the field in the bonds to be

$$\psi_{\mu \beta }^{\left(\alpha \right)}=\phi_{\mu }^{\left(\alpha \right)}\frac{\text{sin}\left[k\left(l_{\mu \beta }-x\right)\right]}{\text{sin}\left[kl_{\mu \beta }\right]}+\phi_{\beta }^{\left(\alpha \right)}\frac{\text{sin}\left[\mathrm{kx}\right]}{\text{sin}\left[kl_{\mu \beta }\right]}$$ (15)

where $k=\frac{\omega }{c}\sqrt{\varepsilon_{r}}$ is the wave number, it is easily seen that this ansatz satisfies the wave equation and the continuity boundary conditions (i.e., when *x* = 0 or *x*=*l*_μβ_). Meanwhile, we can use the current conservation boundary condition to constrain the field amplitudes on the vertices. Since we have

$$\frac{d\psi_{\mu \beta }^{\left(\alpha \right)}}{\mathrm{dx}}{|}_{x=0}=-k\phi_{\mu }^{\left(\alpha \right)}\text{cot}\left[kl_{\mu \beta }\right]+k\phi_{\beta }^{\left(\alpha \right)}\text{csc}\left[kl_{\mu \beta }\right]$$ (16)

and

$$\frac{d\psi_{\mu }^{\left(\alpha \right)}}{\mathrm{dx}}{|}_{x=0}=-\mathrm{ik}\left(I_{\alpha }\delta_{\mu \alpha }-\Gamma_{\mu \alpha }\right)$$ (17)

we can express the current conservation boundary condition at vertex μ

$$\begin{array}{c} \left(\sum_{\beta }-{\left[A\right]}_{\mu \beta }\text{cot}\left[kl_{\mu \beta }\right]\right)\phi_{\mu }^{\left(\alpha \right)}+\sum_{\beta }\text{csc}\left[kl_{\mu \beta }\right]\phi_{\beta }^{\left(\alpha \right)}\\ =\left\{ \begin{array}{c} i\left(I_{\alpha }\delta_{\mu \alpha }-\Gamma_{\mu \alpha }\right),\;\text{if vertex}\;\mu \;\text{has}\;a\;\text{lead connection}\\ 0,\;\mathrm{else} \end{array}\right. \end{array}$$ (18)

which can be immediately expressed in a matrix form for all vertices of the graph

$$M|\phi^{\left(\alpha \right)}\rangle =iW\left[{|s^{+}\rangle }^{\left(\alpha \right)}-{|s^{-}\rangle }^{\left(\alpha \right)}\right]$$ (19)

The components of the wave operator are

$${\left[ℳ\right]}_{\mu \beta }=\left\{ \begin{array}{cc} \sum_{\gamma }-{\left[A\right]}_{\mu \gamma }\text{cot}\left[kl_{\mu \gamma }\right], & \mu =\beta \\ {\left[A\right]}_{\mu \beta }\text{csc}\left[kl_{\mu \beta }\right], & \mu \neq \beta \end{array}\right.$$ (20)

The amplitudes on the vertices are encoded in $|\phi^{\left(\alpha \right)}\rangle \dot{=}{\left[\phi_{1}^{\left(\alpha \right)},\phi_{2}^{\left(\alpha \right)},\ldots \right]}^{T}$, while the incoming and scattered wave amplitudes are encoded in ${|s^{+}\rangle }^{\left(\alpha \right)}\dot{=}{\left[0,\ldots ,\underset{\text{vertex}\;\alpha }{\underbrace{1}},\ldots ,0\right]}^{T}$ and ${|s^{-}\rangle }^{\left(\alpha \right)}\dot{=}{\left[\ldots ,\Gamma_{\mu \alpha },\ldots \right]}^{T}$. The binary filtering matrix **W** provides a connection between the basis of the lead wave amplitudes and the vertex wave amplitudes, such that continuity at vertices where leads are connected is expressed as

$$W^{T}|\phi^{\left(\alpha \right)}\rangle ={|s^{+}\rangle }^{\left(\alpha \right)}+{|s^{-}\rangle }^{\left(\alpha \right)}$$ (21)

Linearity of the problem enables straightforward generalization to arbitrary incoming wavefronts so that we can drop the α superscript,

$$M|\phi \rangle =iW\left[|s^{+}\rangle -|s^{-}\rangle \right];|s^{-}\rangle =-|s^{+}\rangle +W^{T}|\phi \rangle$$ (22)

Inserting the second into the first, the steady-state field can be solved from the input wavefront,

$$|\phi \rangle =2i\text{GW}|s^{+}\rangle ;G\equiv M+iWW^{T}$$ (23)

which can be inserted back into the second of Eq. 22 to obtain the scattering matrix relation

$$|s^{-}\rangle =S|s^{+}\rangle ;S=-I+2iW^{T}\text{GW}$$ (24)

We note that in case of the network model of Fig. 1A consisting of *N* unit cells (and 4*N* vertices) the wave operator $M\equiv M_{N}$.

### Simulation details

The scattering matrix formalism for graphs was used for simulating the slow wave chain for various system sizes to demonstrate how larger arrays can enhance the lower limit of the sublinear response. Simulations were performed with three leads connected at only one side of the system. Losses in the coaxial cables were modeled by introducing an imaginary part to the index of refraction $n_{r}=n_{r}^{\prime }+in_{r}^{\prime \prime }$ such that the wave vector in the cables is $k=\left(\frac{\omega }{c}\right)\cdot n_{r}$ (ω is the angular frequency of the incident wave and *c* is the speed of light in vacuum) via the imaginary part of its wave number such that $\text{Im}\left(k\right)=\gamma \left(A\;\text{Re}\left[k\right]+B\sqrt{\text{Re}\left[k\right]}\right)$, where $A=10^{-3}$ and $B=3.95\times 10^{3}\sqrt{m}$ according to the manufacturer’s cable specification sheet with γ=1. Larger system sizes require less loss to suppress the Fabry-Perot oscillations in the reflection, so to demonstrate the benefit larger arrays can offer, the results of Fig. 1 (C and D) were simulated using γ=0.75 for N=40 unit cells, γ=0.4 for N=100 unit cells, and γ=0.18 for N=200 unit cells. In addition, the input wavefront that was injected in the structure was chosen to be orthogonal to the eigenstate of the reflection operator $S^{†}\left(f_{\text{SIP}}\right)S\left(f_{\text{SIP}}\right)$ corresponding to the minimal eigenvalue. (Recall that in the presence of losses, the **S** matrix is subunitary). For a detailed discussion, see the Supplementary Materials.

### Transfer matrix

The unit cell transfer matrix, $T_{n}$, is an operator that provides a connection to the wave in the ${\left(n+1\right)}^{\mathrm{st}}$ unit cell, given those in the previous unit cells, e.g.,

$$|\varphi_{n+1}\rangle =T_{n}|\varphi_{n}\rangle ;|\varphi_{n}\rangle \dot{\equiv }{\left(|\phi_{n}\rangle \;|\phi_{n-1}\rangle \right)}^{T}$$ (25)

In the case where interactions are restricted between nearest neighbor unit cells, such as in the network in Fig. 1A, the transfer matrix can be formulated directly from the equations of motion, Eq. 5

$$|\phi_{n+1}\rangle =-M_{n,n+1}^{-1}M_{n,n}|\phi_{n}\rangle -M_{n,n+1}^{-1}M_{n,n-1}|\phi_{n-1}\rangle$$ (26)

Therefore, in this representation, the square blocks of the transfer matrix are

$$T_{n}\dot{=}\left(\begin{array}{cc} -M_{n,n+1}^{-1}M_{n,n} & -M_{n,n+1}^{-1}M_{n,n-1}\\ I_{4} & 0 \end{array}\right)$$ (27)

where $I_{4}$ is the 4 × 4 identity matrix. The 4 × 4 matrices $M_{n,m}$ are given by the *m*th and *n*th subblocks of the wave operator in Eq. 20.

The unit cell transfer matrix of periodic structures can be used to formulate Bloch’s theorem as an eigenvalue problem

$$T_{n}\left(\omega \right)|\varphi \rangle =e^{\mathrm{iqa}}|\varphi \rangle$$ (28)

Generically, EPDs in the spectrum of $T_{n}$ occur simultaneously with stationary points in the Bloch dispersion relation of the corresponding order. Specifically, an SIP forms in the Bloch dispersion relation when three Bloch eigenmodes coalesce.

## Supplementary Materials

### This PDF file includes:

Supplementary Text Fig. S1 References
