# Supplementary material for: Noise-resilient exceptional point sensing with immunity to undesired perturbations
Source: Sci Adv. 2026 Feb 27;12(9):eaeb7018. doi: 10.1126/sciadv.aeb7018 (PMC12947881; doi:10.1126/sciadv.aeb7018)
Supplement: Supplementary file 1 — Supplementary Text Fig. S1 References [file sciadv.aeb7018_sm.pdf]

Science Advances
AAAS

Supplementary Materials for

Noise-resilient exceptional point sensing with immunity to undesired perturbations

Serena Landers *et al.*

Corresponding author: Tsampikos Kottos, tkottos@wesleyan.edu

Sci. Adv. **12**, eaeb7018 (2026)
DOI: 10.1126/sciadv.aeb7018

This PDF file includes:

Supplementary Text
Fig. S1
References

Supplementary Text

Reflectance signal near SIP under ideal and non-ideal conditions and practical considerations for the calibration of measurand

Under ideal conditions, i.e., zero losses, semi-infinite periodic structures, and appropriate incident wavefront (see also below), the SIP manifests itself in the reflectance spectrum as a cusp at f_{SIP} (see for example Refs. (*33*, *35*, *48*, *49*)). However, losses wash out the reflectance cusp at $f = f_{SIP}$ (see Ref. (*39*)) while small system sizes N "pollute" the reflected signal with backward propagating Bloch modes and evanescent modes excited by the opposite sample interface (see Ref. (*38*)). All these features affect the sublinear scaling of the reflectance for very small detuning $\nu < \nu^*$. Instead, the sublinear scaling law is maintained for larger detuning $\nu^* < \nu$.

In our sensing protocol, we have considered such wash-out effects in the extraction of the reference reflectance, R_0, that we have used in the definition of $\Delta R(\nu) \equiv |R(f_{SIP} + \nu) - R_0|$. Specifically, we have fit the experimental/computational reflectance data with the functional behavior $R(f) = R_0 + \alpha|f - f_{SIP}|^{\beta}$, which is applicable to a frequency range $f \in [f_{SIP} - \nu_c^-, f_{SIP} - \nu^*]$ or $f \in [f_{SIP} + \nu^*, f_{SIP} + \nu_c^+]$. In practice, the fitting range has been always chosen to be on the left side (smaller frequencies) of f_{SIP}, i.e., $f \in [f_{SIP} - \nu_c^-, f_{SIP} - \nu^*]$ where the differential reflectance consistently demonstrated (both in simulated data and in measurements) a larger frequency range over which the sublinear scaling is applicable. During the fitting process, the parameters f_{SIP}, β, were given a tolerance around their expected values $f_{SIP}^{exp}, \beta^{exp}$ which was allowed to be less than 10%. The expected SIP frequency f_{SIP}^{exp} was estimated from the computational/experimental data to be the frequency for which $\left.\frac{dR(f)}{df}\right|_{f=f_{SIP}^{exp}} = 0$, while the expected exponent is set by default to be $\beta^{exp} = 2/3$. An example of the fitting process is shown in Fig. R1 below. The extracted constant $R_0 \neq R(f_{SIP})$ is then used in the evaluation of $\Delta R(\nu)$. Notice that once R_0 has been extracted, the measurand $\Delta R(\nu)$ is uniquely determined for the specific scattering sample that is used as SIP-based sensing platform (essentially R_0 acts as a unique calibration point for the specific platform that considers the non-ideal conditions). Other fitting schemes have provided qualitatively similar results. The plateau of the differential reflectance in the $\nu \to 0$ limit reflects the fact that $\Delta R(\nu = 0) = |R(f_{SIP}) - R_0| \neq 0$.

To further confirm that the sublinear law has its origin in the anomalous properties of the SIP (and the underlying EPD-3), we have analyzed the scaling of $\Delta R(\nu)$ for increasingly larger system sizes. In this case, we expect (see also Fig. 3c in Ref. (*33*)) a stretch of the sublinear scaling range (and therefore an enhancement of the dynamical range of the SIP sensor) towards smaller values of detuning ν (i.e. smaller perturbations). A consequence of this stretching is that $\Delta R(\nu \to 0) \to 0$ as we approach the thermodynamical limit corresponding to $N \to \infty$ while losses become smaller such that the ratio of the system size to the absorption length remains approximately constant. A scheme to approach the thermodynamic limit is provided by realizing that the absorption length for a typical propagating mode inside the structure is given by $\xi_p \sim 1/\gamma$, where γ is the loss coefficient that characterizes the structure. Instead, for efficiently excited slow light the absorption length is $\xi_s \sim 1/\sqrt[3]{\gamma}$ (*39*). In order to avoid contamination of the reflected signal due to back-

reflection from the opposite surface of the finite sample, one requires $\max\{\xi_s, \xi_p\} \leq N$. For small values of loss coefficient (the case of interest) $\xi_s < \xi_p$ and therefore $N/\xi_p \sim O(1) \rightarrow N \cdot \gamma \sim O(1)$ defines the appropriate thermodynamic limit that allows us to maintain reflected signal that is sanitized from back-reflections from the opposite surface.

The results of such analysis are shown in Fig. 1D of the main text.

Wavefront shaping for finite structures

For lossless, semi-infinite SIP-structures, the optimal incident wavefront, $|r_o\rangle$, that results in the sublinear scaling of the differential reflectance must avoid exciting a particular linear combination, $|t_o\rangle$, of the slow propagating and evanescent Bloch modes of the SIP-structure. This combination enforces continuity of the waves at the interface via destructive interference, which enables efficient energy conversion into the SIP structure, but destroys the sublinear response in the differential reflectance. Instead, the optimal wavefront $|r_o\rangle$ can be evaluated in terms of the interfacial scattering matrix (including both propagating and evanescent channels) by enforcing zero evanescent mode excitation (*33, 36*). Such constraint leads to a maximum reflectance from the SIP-structure.

In finite, weakly lossy structures, the wavefront that maximizes the reflectance is naturally associated with the reflection eigenvector that corresponds to the maximum eigenvalue of the reflectance operator $\mathbf{S}^{\dagger}\mathbf{S}$. Conversely, the eigenvector associated with the smallest reflection eigenvalue efficiently excites the analogue of $|t_o\rangle$ in the finite structure, thereby suppressing the SIP sublinear response of the differential reflectance.

In our case the smallest reflection eigenvalue of the 3×3 reflection operator $\mathbf{S}^{\dagger}\mathbf{S}$, is orders of magnitude smaller than the second and third eigenvalues which are of the same order. The eigenvectors associated with the latter eigenvalues are practical finite-N approximations of $|r_o\rangle$, which couple inefficiently to the analogue of $|t_o\rangle$, producing the characteristic sublinear response $\Delta R \sim \nu^{2/3}$. Accordingly, we have used the second eigenvector in our finite-N scaling analysis, because it provided the clearest and most stable $\nu^{2/3}$ scaling. The finite-N approximation nature of $|r_o\rangle$, which partially excites the analogue of $|t_o\rangle$, may further distort the shape of the cusp, e.g., reflectance value, concavity. Finally, the reflectance cusp is washed out extremely close to f_{SIP}, primarily due to losses. However, this washout can be pushed to progressively smaller detuning as N increases and losses are reduced such that the ratio of the sample length to the absorption length of the propagating modes is held approximately constant.

In our experiments (due to limited sources) we have used a simple wavefront associated with an incident wave from only one port. While such wavefront is not equal to any of the reflection eigenvectors, the sublinear response of the differential reflectance is well maintained. Therefore, one concludes that such injected wavefront inefficiently excites the analogue of $|t_o\rangle$.

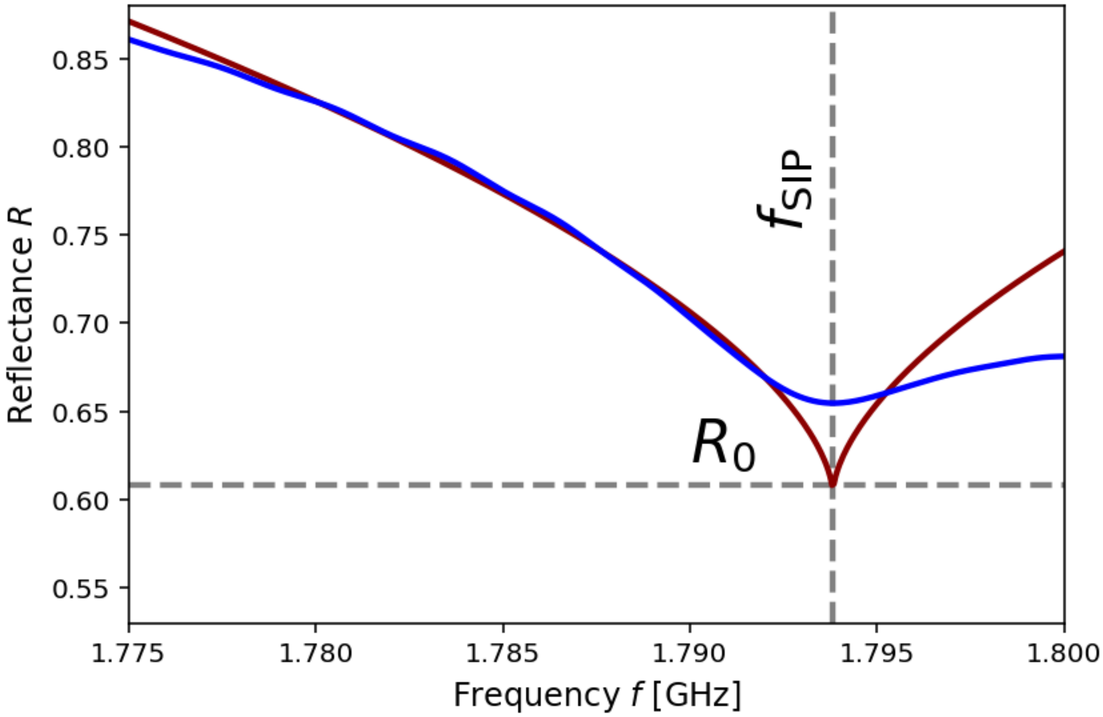

Fig. S1. Practical approach for extracting reference reflectance. An example for the fitting process to extract the reference reflectance $\boldsymbol{R_0}$ used in our evaluation of $\boldsymbol{\Delta R(\nu)}$. The system consists of $\boldsymbol{N = 40}$ periods with modeled loss strength being $\boldsymbol{\gamma = 0.75}$ (for simulation details see "Simulation Details" section in the Materials and Methods). The red line indicates the best fit with the functional form $\boldsymbol{R(f) = R_0 + \alpha|f - f_{SIP}|^{\beta}}$. The extracted $\boldsymbol{R_0}$ is indicated with a dashed horizontal line. The asymmetry of the reflectance spectrum for negative and positive detuning values $\boldsymbol{\nu}$ has its origin in the non-ideal conditions, i.e., present of losses, finite structure, and non-perfect injected wavefront (see Ref. (*49*) and specifically Figs. 7a,b,c for non-ideal wavefront and Fig. 7d for ideal wavefront). To this end, we always choose to fit our experimental/computational data in the negative detuning regime where we consistently observe a better agreement with the SIP-expected 2/3 sublinear scaling.

REFERENCES

1. R. S. Cassel, W. G. Tobias, B. L. Marlow, Quantum vs. classical complementary PNT, The MITRE Corporation (2023).

2. F. Dell'Olio, T. Tatoli, C. Ciminelli, M. Armenise, Recent advances in miniaturized optical gyroscopes. *J. Eur. Opt. Soc.* **9**, 14013 (2014).

3. H. C. Lafevre, *The Fiber-Optic Gyroscope*, (Ariech House, ed. 2, 2014).

4. M. N. Armenise, C. Ciminelli, F. Dell' Olio, V. M. N. Passaro, *Advances in Gyroscope Technologies* (Springer, 2010).

5. I. Lavrinovica, J. Judvaitis, D. Laksis, M. Skromule, K. Ozols, A comprehensive review of sensor-based smart building monitoring and data gathering techniques. *Appl. Sci* **14**, 10057 (2024).

6. B. Santos, A. Soares, T.-A. Nguyen, D.-K. Min, J.-W. Lee, F.-A. Silva, IoT sensor networks in smart buildings: A performance assessment using queuing models. *Sensors* **21**, 5660 (2021)

7. M. De Carlo, F. De Leonaridis, R. A. Soref, L. Colatorti, V. M. N. Passaro, Non-Hermitian sensing in photonics and electronics: A review. *Sensors* **22**, 3977 (2022)

8. J. Wiersig, Prospects and fundamental limits in exceptional point-based sensing. *Nat. Comm.* **11**, 2454 (2020).

9. J. Wiersig, Review of exceptional point-based sensors. *Photon. Res.* **8**, 1457 (2020).

10. J. Wiersig, Enhancing the sensitivity of frequency and energy splitting detection by using exceptional points: Application to microcavity sensors for single-particle detection. *Phys. Rev. Lett.* **112**, 203901 (2014).

11. M.-A. Miri, A. Alù, Exceptional Points in optics and photonics. *Science* **363**, 7709 (2019).

12. Ş. K. Özdemir, S. Rotter, F. Nori, L. Yang, Parity–time symmetry and exceptional points in photonics. *Nat. Mater.* **18**, 783–798 (2019)

13. R. El-Ganainy, K. G. Makris, M. Khajavikhan, Z. H. Musslimani, S. Rotter, D. N. Christodoulides, Non-Hermitian physics and PT symmetry. *Nat. Phys.* **14**, 11–19 (2017).

14. L. Feng, R. El-Ganainy, L. Ge, Non-Hermitian photonics based on parity–time symmetry. *Nat. Photon.* **11**, 752–762 (2017).

15. H. Hodaei, A. U. Hassan, S. Wittek, H. Garcia-Gracia, R. El-Ganainy, D. N. Christodoulides, M. Khajavikhan, Enhanced sensitivity at higher-order exceptional points. *Nature* **548**, 187–191 (2017).

16. W. Chen, Ş. Kaya Özdemir, G. Zhao, J. Wiersig, L. Yang, Exceptional points enhance sensing in an optical microcavity. *Nature* **548**, 192–196 (2017).

17. M. P. Hokmabadi, A. Schumer, D. N. Christodoulides, M. Khajavikhan, Non-Hermitian ring laser gyroscopes with enhacned Sagnac sensitivity. *Nature* **576**, 70–74 (2019).

18. Y.-H. Lai, Y.-K. Lu, M.-G. Suh, Z. Yuan, K. Vahala, Observation of the excpetional-point-enhanced Sagnac effect. *Nature* **576**, 65–69 (2019).

19. Y.-H. Lai, M.-G. Suh, Y.-K. Lu, B. Shen, Q.-F. Yang, H. Wang, J. Li, S. H. Lee, K. Y. Yang, K. Vahala, Earth rotation measured by a chip-scale ring laser gyroscope. *Nat. Photon.* **14**, 345–349 (2020).

20. R. Kononchuk, T. Kottos, Orientation-sensed optomechanical accelerometers based on exceptional points. *Phys. Rev. Res.* **2**, 023252 (2020)

21. R. Kononchuk, J. Z. Cai, F. Ellis, R. Thevamaran, T. Kottos, Exceptional-point-based accelerometers with enhanced signal-to-noise ratio. *Nature* **607**, 697–702 (2022).

22. Z. Dong, Z. Li, F. Yang, C.-W. Qiu, J. S. Ho, Sensitivity readout of implantable micro sensors using a wireless system locked to an exceptional point. *Nat. Electron.* **2**, 335–342 (2019)

23. J. Li, F. Zhang, X. Xia, K. Zhang, J. Wu, Y. Liu, C. Zhang, X. Cai, J. Lu, L. Xu, R. Wan, D. Hazarika, W. Xuan, J. Chen, Z. Cao, Y. Li, H. Jin, S. Dong, S. Zhang, Z. Ye, M. Yang, P.-Y. Chen, J. Luo, An ultrasensitive multimodal intracranial pressure biotelemetric system enabled by exceptional point and iontronics. *Nat. Comm.* **15**, 9557 (2024).

24. X. Lu, Y. Yuan, F. Chen, X. Hou, Y. Guo, L. Reindl, Y. Fu, W. Luo, D. Zhao, Harnession excpetional points for ultrahigh sensitive acoustic wave sensing. *Microsyst. Nanoeng.* **11**, 44 (2025).

25. Z. Xiao, H. Li, T. Kottos, A. Alù, Enhanced sensing and nondegraded thermal noise performance based on PT-symmetric electronic circuits with sixth-order exceptional point. *Phys. Rev. Lett.* **123**, 213901 (2019).

26. H. Wang, Y.-H. Lai, Z. Yuan, M.-G. Suh, K. Vahala, Petermann-factor sensitivity limit near an exceptional point in a Brilluin ring laser gyroscope. *Nat. Commun.* **11**, 1610 (2020).

27. H.-K. Lau, A. A. Clerk, Fundamental limits and non-reciprocal approach in non-Hermitian quantum sensing. *Nat. Comm.* **9**, 4320 (2018)

28. W. Langbein, No exceptional precision of exceptional-point sensors. *Phys. Rev. A* **98**, 023805 (2018).

29. J. Wiersig, Robustness of exceptional point-based sensors against parametric noise: The role of Hamiltonian and Liouvillian degeneracies. *Phys. Rev. A* **101**, 053846 (2020).

30. H. Loughlin, V. Sudhir, Exceptionall-point sensors offer no fundamental signal-to-noise ratio enhancement. *Phys. Rev. Lett.* **132**, 243601 (2024).

31. J. Naikoo, R. W. Chhajlany, J. Kołodyński, Multiparameter estimation perspective on non-Hermitian singularity-enhanced sensing. *Phys. Rev. Lett.* **131**, 220801 (2023).

32. W. Ding, X. Wang, S. Chen, Fundamental sensitivity limits for non-Hermitian quantum sensors. *Phys. Rev. Lett.* **131**, 160801 (2023).

33. W. Tuxbury, R. Kononchuk, T. Kottos, Non-resonant exceptional points as enablers of noise-resilient sensors. *Commun. Phys.* **5**, 210 (2022).

34. A. Figotin, I. Vitebskiy, Slow wave phenomena in photonic crystals. *Laser Photon. Rev.* **5**, 201–213 (2011)

35. A. Figotin, I. Vitebskiy, Slow light in photonic crystals. *Waves Random Complex Media* **16**, 293 (2006).

36. N. Gutman, C. M. de Sterke, A. A. Sukhorukov, L. C. Botten, Slow and frozen light in optical waveguides with multiple gratings: Degenerate band edges and stationary inflection points. *Phys. Rev. A* **85**, 033804 (2012)

37. A. Herrero-Parareda, I. Vitebskiy, J. Scheuer, F. Capolino, Frozen mode in an asymmetric serpentine optical waveguide. *Adv. Photon. Res.* **3**, 2100377 (2022).

38. H. Li, I. Vitebskiy, T. Kottos, Frozen mode regime in finite periodic structures. *Phys. Rev. B* **96**, 180301(R) (2017).

39. W. Tuxbury, L. J. Fernandez-Alcazar, I. Vitebskiy, T. Kottos, Scaling theory of absorption in the frozen mode regime. *Opt. Lett.* **46**, 3053–3056 (2021).

40. Z. M. Gan, H. Li, T. Kottos, Effects of disorder in frozen-mode light. *Opt. Lett.* **44**, 2891–2894 (2019)

41. S. Landers, W. Tuxbury, I. Vitebskiy, T. Kottos, Robust nonlinear isolators based on frozen mode exceptional point degeneracies. *Phys. Rev. Res.* **7**, 013110 (2025)

42. S. Landers, W. Tuxbury, I. Vitebskiy, T. Kottos, Unidirectional amplification in the frozen mode regime enabled by a nonlinear defect. *Opt. Lett.* **49**, 4967–4970 (2024)

43. N El-Sheimy, H. Hou, X. Niu, Analysis and modeling of inertial sensors using Allan variance. *IEEE Trans. Instrum. Meas.* **57**, 140 (2008)

44. A. E. E. Wallin, Allan Tools, (2019) [Online] Available at: https://github.com/aewallin/allantools

45. A. Suntharalingam, L. Fernández-Alcázar, R. Kononchuk, T. Kottos, Noise resilient exceptional-point voltmeters enabled by oscillation quenching phenomena. *Nat. Commun.* **14**, 5515 (2023).

46. N. Furman, A. Herrero-Parareda, A. Rapp, I. Vitebskiy, R. Gibson, B. Thompson, D. Brown, R. Bedford, F. Capolino, Dispersion engineering of periodic silicon photonic waveguides for enhanced group delay in the frozen mode. *Opt. Mater. Express* **15**, 2743–2758 (2025)

47. T. Baba, H. Nguyen, N. Yazawa, Y. Terada, S. Hashimoto, T. Watanabe, Slow-light Mach–Zehnder modulators based on Si photonic crystals. *Sci. Technol. Adv. Mater.* **15**, 024602 (2014)

48. J. Ballato, A. Ballato, A. Figotin, I. Vitebskiy, Frozen light in periodic stacks of anisotropic layers. *Phys. Review E* **71**, 036612 (2005)

49. A. Figotin, I. Vitebskiy, Electromagnetic unidirectionality in magnetic photonic crystals. *Phys. Review B* **67**, 165210 (2003)
